# Autophagy and Microglia: Novel Partners in Neurodegeneration and Aging

**DOI:** 10.3390/ijms18030598

**Published:** 2017-03-09

**Authors:** Ainhoa Plaza-Zabala, Virginia Sierra-Torre, Amanda Sierra

**Affiliations:** 1 Achucarro Basque Center for Neuroscience, 48170 Zamudio, Spain; virginiasierra1@gmail.com; 2 Department of Neurosciences, University of the Basque Country EHU/UPV, 48940 Leioa, Spain; 3 Ikerbasque Foundation, 48013 Bilbao, Spain

**Keywords:** microglia, autophagy, phagocytosis, inflammation, aging, neurodegeneration

## Abstract

Autophagy is emerging as a core regulator of Central Nervous System (CNS) aging and neurodegeneration. In the brain, it has mostly been studied in neurons, where the delivery of toxic molecules and organelles to the lysosome by autophagy is crucial for neuronal health and survival. However, we propose that the (dys)regulation of autophagy in microglia also affects innate immune functions such as phagocytosis and inflammation, which in turn contribute to the pathophysiology of aging and neurodegenerative diseases. Herein, we first describe the basic concepts of autophagy and its regulation, discuss key aspects for its accurate monitoring at the experimental level, and summarize the evidence linking autophagy impairment to CNS senescence and disease. We focus on acute, chronic, and autoimmunity-mediated neurodegeneration, including ischemia/stroke, Alzheimer’s, Parkinson’s, and Huntington’s diseases, and multiple sclerosis. Next, we describe the actual and potential impact of autophagy on microglial phagocytic and inflammatory function. Thus, we provide evidence of how autophagy may affect microglial phagocytosis of apoptotic cells, amyloid-β, synaptic material, and myelin debris, and regulate the progression of age-associated neurodegenerative diseases. We also discuss data linking autophagy to the regulation of the microglial inflammatory phenotype, which is known to contribute to age-related brain dysfunction. Overall, we update the current knowledge of autophagy and microglia, and highlight as yet unexplored mechanisms whereby autophagy in microglia may contribute to CNS disease and senescence.

## 1. Introduction

Autophagy is emerging as one of the core orchestrators of healthy aging [1,2]. This self-degradation process is present in all mammalian cells and tissues, including the central nervous system (CNS), and specializes in directing unnecessary or damaged intracellular material to the lysosome, the major cellular organelle that digests and recycles all types of macromolecules. Autophagy, as a constitutive mechanism, participates in the basal turnover of long-lived proteins and organelles, playing a major role as a checkpoint for quality control. On the other hand, stressful situations such as metabolic starvation or functional damage induce an adaptive autophagic response to restore cellular homeostasis. Thus, adaptive autophagy provides the cell with nutrients and energy during metabolic shortage and relieves it from toxic components during functional damage. Accordingly, a correct completion of the autophagic response is central for optimal CNS physiology and the promotion of neuronal survival. This is evidenced by the elevated number of connections made between the dysregulation of autophagy, aging, and neurodegeneration.

In this review, we will describe the role of autophagy (dys)regulation in the aged and diseased brain. Particularly, we will focus on microglia, the brain’s resident macrophages with intrinsic capability to respond to CNS damage, promoting repair and a correct brain function. First, we will briefly outline the process of autophagy and its regulation, and summarize key technical aspects for the correct monitoring of autophagy at the experimental level. Then we will review the role of autophagy in neurons and the impact of autophagy failure in neurodegeneration. Finally, we will detail the current state of the literature on the role of autophagy in peripheral macrophages and microglia, including the regulation of phagocytosis and the inflammatory response.

## 2. Autophagy: The Facts

Three types of autophagy have been described depending on substrate selectivity and the pathway used to deliver the cargo to lysosomes: chaperone-mediated autophagy, microautophagy, and macroautophagy. These processes have been detailed in excellent reviews [3,4,5,6] and here we will focus on macroautophagy, since it is the autophagic pathway that has mainly been connected to brain aging and pathology. Macroautophagy (hereafter referred to as autophagy) is a dynamic process that delivers degradation substrates to lysosomes forming an intermediate structure called the autophagosome or the autophagic vacuole. The signaling pathways that lead to the initiation of the autophagic cascade are varied and complex and have been reviewed elsewhere [7]. Briefly, autophagy is regulated by intracellular and extracellular nutrient and damage sensors that most often converge on the mechanistic target of rapamycin complex 1 (MTORC1) [8], a central suppressor of autophagy. During canonical autophagy the inhibition of MTORC1 allows the activation of the unc-51 like autophagy activating kinase 1 (ULK-1) containing autophagy pre-initiation complex. Subsequently, the beclin-1/vacuolar protein sorting 34 (BECN-1/Vps34) complex is recruited to catalyze the formation of phosphatidyl-inositol-3-phosphate (PI3P) [9], necessary for the formation of the preautophagosomal membrane or phagophore. The elongation of this structure is regulated by the sequential and coordinated action of autophagy-related (ATGs) proteins, leading to the formation of the autophagosome [9,10], the double-membrane bound vesicle that sequesters autophagic cargo on its lumen. Hence, ATG-3, ATG-4, and ATG-7 catalyze the lipidation of microtubule-associated light chain 3 (LC3), which accumulates in autophagosomal membranes and mediates cargo engulfment by binding to autophagic substrates. Additionally, ATG-7 together with ATG-10 supports the formation of ATG-5/ATG-12/ATG-16L1 complex, enabling the elongation of the autophagosome membrane. Once formed, autophagosomes are actively transported through the cytosol over microtubules where they mature and become acidified by interacting with the endosomal compartment [11,12]. Finally, once they reach the perinuclear region where lysosomes are enriched, autophagosomes fuse with these acidic organelles, forming the autophagolysosome. This degradative compartment contains all the necessary enzymatic machinery to digest cell-derived cargo to essential molecules and recycle them back into the cytoplasm.

The selectivity of the autophagic response depends on the cellular context and the inducing stimulus. Hence, when autophagic substrates such as misfolded/aggregated proteins or damaged organelles accumulate, autophagy selectively recognizes the toxic cargo and targets it to the lysosome for degradation. This selectivity is achieved by a varied pool of autophagic receptors (e.g., Sequestosome-1 (SQSTM-1 or p62), neighbor of BRCA1 (NBR1), NIP-3 like protein X (NIX), and others) that act as a bridge between the autophagosome and the autophagic substrate [13]. Autophagic receptors contain an LC3-interacting motif (which allows them to bind to the autophagosomal membrane) as well as a motif that recognizes specific degradation tags such as poly-ubiquitin chains in autophagic cargo. Selective autophagy has been described for mitochondria, proteotoxic aggregates, and intracellular pathogens, among others, referred to as mitophagy, aggrephagy, and xenophagy, respectively. In contrast, non-selective autophagy occurs when autophagic products such as essential nutrients are needed to maintain metabolic balance and proper cellular function (e.g., nutrient starvation). In this case, relatively non-selective mechanisms lead to the engulfment of whole cytoplasmic portions, which are digested in the lysosome to obtain nutrients and energy.

Physiological levels of autophagy are essential for the promotion of cytoprotection and survival in response to a variety of stressful situations such as starvation [14], proteotoxicity and organelle damage [15], and infection [16]. Nevertheless, a long-standing and controversial question is whether excessive autophagy may mediate cell death under certain circumstances. Historically, autophagic cell death was described as a form of programmed cell death, together with apoptosis [17]. Nevertheless, the assessment of autophagic cell death was based on morphological observations correlating the abundance of autophagic structures during certain forms of cell death [18], without assessing the functional contribution of autophagy to the death process. Later work challenged this concept, raising the question of whether autophagy induction was, rather, an unsuccessful attempt to promote cell survival [18]. Indeed, the Nomenclature Committee on Cell Death (NCCD) revised the term autophagic cell death, recommending its use exclusively when genetic or pharmacological inhibition of autophagy prevented or limited cell demise [19]. Therefore, although autophagy mediates death in specific cellular contexts and subpopulations such as developing tissue or when apoptosis is compromised, the overall contribution of autophagy to cell death does not seem to be substantial [17].

In summary, autophagy is a multi-step process that needs to be carefully assessed at the experimental level since its dynamics after cellular or tissue insult change in a time-dependent fashion. Thus, in a specific pathological setting autophagy may play a cytoprotective, survival promoting role at early time points, whereas under unresolved damage the long-term induction of autophagy may lead to detrimental flux dysregulations that end with apoptotic or necrotic cell death [20,21].

## 3. Technical Pitfalls in Autophagy Research

The experimental monitoring of autophagy is complex since its fulfillment depends on the sequential formation of transitory structures (phagophore, autophagosome, autophagolysosome), which are usually short-lived, before their degradation in the lysosome. Indeed, assessing the number or volume of specific autophagic elements at a single time point is not an accurate measure of autophagic activity [22]. For instance, the accumulation of autophagosomes or their biochemical markers (such as LC3 or other ATG proteins), which is frequently used as a measure of autophagic activity, may reflect either that autophagy is in fact activated or that the autophagy flux is blocked. Autophagy flux or the lysosomal clearance of autophagosomes may be blocked due to (1) disruptions in the cellular transport of these structures; (2) their fusion with lysosomes; and/or (3) to a deficient lysosomal compartment. To avoid this type of misconception, standardized procedures for the use and interpretation of autophagy assays have been published in open access and are periodically updated [22]. These guidelines, which have been prepared by the international autophagy research community, strongly recommend the use of a combination of complementary experimental techniques and approaches. Among other interventions, the use of pharmacological agents such as lysosomal inhibitors enables an accurate measurement of the complete autophagy flux (that is, the correct formation of autophagosomes as well as their proper fusion with functional lysosomes). Nevertheless, it is still common to find studies that do not measure the overall flux through the autophagic pathway, thereby reporting contradictory results and complicating the interpretation of data.

Another caveat in autophagy research is the limited selectivity of pharmacological agents that modulate autophagy. Indeed, commonly used autophagy regulators such as 3-methyladenine (3-MA) (phosphatidyl-inositol-3 kinase (PI3K) inhibitor; prevents autophagosome formation), Bafilomycin-A1 (vacuolar-ATPase inhibitor; enhances lysosomal pH blocking lysosomal degradation), and Rapamycin (enhances autophagy flux inhibiting MTORC1) usually have dose- and time-dependent off-target effects [22]. To overcome the limitations of these pharmacological approaches, genetic silencing or enhancing of key autophagic proteins such as ATGs is also recommended [22]. Nonetheless, recent work has also highlighted a role for ATG proteins beyond autophagy. For instance, calpain-cleaved ATG-5 translocates to mitochondria and participates in apoptosis induction [23]. Similarly, ATGs including ATG-5, BECN-1 (ATG-6), and ATG-7 are involved in LC3 (ATG-8) lipidation to phagosomes and other macroendocytic vacuole membranes, where they mediate extracellular substrate turnover in the lysosome [24]. Finally, and taking into consideration the limitations specified above, the impact of autophagy in rodent models of aging and neurodegeneration has been hindered by the embryonic or early post-natal lethality of constitutive knockouts of essential autophagy genes [25,26]. Nevertheless, the development of tissue- or cell-specific conditional knockouts in rodents [27,28] has significantly advanced our understanding of the role of autophagy in age-related physiological and pathological settings at the organism level. In conclusion, the experimental analysis of autophagy in cultured cells as well as animal models needs to be carefully addressed, using complementary techniques that enable the accurate measurement of the functional status of this dynamic process through time. Given the complexity of the experimental monitoring of autophagy, in this review we will differentiate between studies that have provided evidence to estimate the complete autophagy flux from work that has described a role for specific proteins and/or genes of the autophagic machinery.

## 4. Autophagy in the Central Nervous System (CNS)

In the CNS, autophagy has mainly been studied in neurons, where it plays a crucial role as a checkpoint for protein and organelle quality control. Indeed neurons, as post-mitotic cells, cannot dilute the effect of the accumulation of toxic molecules or damaged organelles through cellular division and essentially rely on basal levels of autophagy for survival. Thus, the specific deletion of pivotal autophagic genes such as ATG-7 [28] or ATG-5 [27] from the neural lineage induces the formation of cytoplasmic inclusions and neurodegeneration in the absence of any other pathological event. As such, mounting evidence indicates that autophagy in the CNS plays a major role in the promotion of neuronal health and survival. On the next section, we will briefly summarize the evidences linking loss of autophagic function to CNS senescence and aging, as well as highlight some examples of the contribution of autophagy flux dysregulation to the following neurodegenerative diseases: ischemia/stroke, Alzheimer’s, Parkinson’s, and Huntington’s diseases, and multiple sclerosis.

### 4.1. Aging

Aging is associated with a loss of proteostasis, altered nutrient sensing, organellar and mitochondrial damage, cellular senescence, and stem cell exhaustion, among other dysfunctions [29]. Many of these alterations may arise, at least in part, from deterioration of basal autophagy and/or impairment of correct autophagy flux induction during cellular stress [2], although data assessing the role of autophagy in brain physiological aging is only beginning to emerge. In the human aged brain, a downregulation of autophagy genes including ATG-5, ATG-7, and BECN-1 has been observed [30], and brains of aged rodents exhibit increased MTORC1 activity together with a decline of ATG protein levels [31,32], suggesting decreased autophagic function. Similarly, mice carrying neural lineage-specific deletions of ATG-5 [27] and ATG-7 [28] show spontaneous and accelerated neurodegeneration. As such, the restoration or promotion of autophagic function has been proposed as one possible approach to delay aging, including brain aging [1,33]. Potential strategies include physiologic and pharmacological inducers of autophagy flux, caloric restriction (CR), physical exercise, and MTORC1 inhibitors such as rapamycin, which extend life and health span in many animal models [1,2,34]. Particularly, CR promotes healthy longevity in rhesus monkeys [35,36], where it also reduces brain atrophy [35], although the possible functional impact of CR on primate cognition has not been assessed yet. In agreement, CR significantly prevents the downregulation of several autophagy effectors in aged rodents, which has been correlated with an attenuation of learning and memory deficits [32]. Physical exercise has also been shown to affect autophagy. Resistance exercise enhances autophagy flux in peripheral blood mononuclear cells of aged subjects [37], and acute exercise increases some autophagy markers in the cerebral cortex of mice [38]. However, a direct correlation between physical exercise-stimulated autophagy flux in the brain and the prevention of age-associated cognitive deficits is still missing. In summary, accumulating evidence indicates that a loss of proficient autophagic function may underlie some of the aging-associated defects. Therefore, the promotion of autophagy flux may prove beneficial to delay brain aging.

### 4.2. Acute Neurodegeneration—Ischemia/Stroke

Ischemic stroke is among the most frequent causes of death and permanent disability in the elderly population [39]. It is associated with compromised blood supply to the brain, which translates into different degrees of severity in the affected region, where part of the parenchyma undergoes immediate death (the core) and part becomes partially injured with the potential to recover (the penumbra). Autophagic structures and/or markers are consistently observed in brain tissue derived from rodent models of acute hypoxic/ischemic brain injury [40,41]. However, controversy exists as to whether autophagy plays a protective or toxic role since both the positive [42,43] and the negative [41,43] modulation of autophagy has been reported to promote neuroprotection using in vivo models of brain ischemia. For instance, genetic silencing of Tuberous Sclerosis Complex 1 (TSC-1), which results in MTORC1 activation and the inhibition of autophagy, exacerbates neuronal injury using in vitro and in vivo models of transient global ischemia [44]. Particularly, in vitro downregulation of TSC-1 induces a blockade of the autophagy flux that is prevented by the genetic overexpression of TSC-1, which enhances neuronal survival through the restoration of the autophagic flux [44], suggesting a neuroprotective role for autophagy in brain ischemia. In line with this idea, ischemic preconditioning or the induction of moderate ischemic stress that protects from a subsequent ischemic insult, elicits neuroprotective effects through the induction of sub-lethal endoplasmic reticulum stress and autophagy [40,45]. Moreover, ischemia-reperfusion activates mitophagy in vitro and in vivo, and pharmacological inhibitors of autophagy and mitophagy such as 3-MA and mitochondrial division inhibitor-1 (mdivi-1), respectively, exacerbate neuronal and brain tissue damage [46], suggesting that mitochondrial clearance through autophagy is neuroprotective during the reperfusion phase. In contrast, another study in which BECN-1 was genetically silenced in adult rats showed attenuated infarct volume, tissue injury, and neurological deficits in a transient middle cerebral artery occlusion (tMCAO) model [47]. However, autophagic flux, and the effects of BECN-1 silencing on the autophagic flux were not assessed in this study and therefore it is not possible to conclude whether autophagy inhibition is neuroprotective. In the context of adult hypoxic/ischemic brain injury, additional experimental work is needed to draw solid conclusions on the potential cytoprotective or cytotoxic role of autophagy.

### 4.3. Chronic Neurodegeneration—Alzheimer’s, Parkinson’s, and Huntington’s Diseases

Alzheimer’s Disease (AD) is the most prevalent neurodegenerative disease and is associated with a progressive cognitive decline. Neuropathologically, AD is characterized by synaptic loss, the deposition of extracellular amyloid-β (Aβ) plaques, and the presence of intracellular neurofibrillary tangles containing hyperphosphorylated tau protein. During the last decades, extensive research has focused on AD, and a plethora of mechanisms including neuroinflammation [48], defects in mitochondrial dynamics and function [49], and autophagy failure [50] have been proposed to contribute to AD neurodegeneration. A pathological accumulation of autophagosomes has been observed in neocortical biopsies of AD patients [51], which has been attributed to the aberrant transport and/or acidification of autophagic vacuoles, in turn preventing their efficient clearance of lysosomes [51,52]. Presenilin-1 (PS-1) mutations, which cause early-onset AD, impair lysosomal acidification and induce a blockade of the autophagy flux in fibroblasts of AD patients [53]. Moreover, restoring lysosomal proteolytic activity by deletion of cystatin b, an endogenous inhibitor of certain lysosomal cysteine proteases of the cathepsin family, rescues autophagy flux blockade in neurons, diminishes Aβ load, and prevents learning and memory deficits in an amyloid precursor protein (APP)-overexpressing TgCRND8 mouse model of AD [54]. Although this latter study suggests that autophagy participates in the degradation of Aβ, ATG-7 deletion in forebrain excitatory neurons in APP overexpressing mice (APP23 model) reduces extracellular Aβ plaque deposition at the expense of eliciting an intracellular accumulation of Aβ [55], indicating that autophagy may also play a role in Aβ secretion. Additionally, amyloidosis exacerbates ATG-7 deficiency-induced neurodegeneration and memory impairments [55], which suggests that a lack of neuronal autophagy may aggravate AD pathology. Finally, enhancing autophagy flux by the disaccharide trehalose in the brain of a transgenic P301S human tauopathy mouse model decreases the number of neurons containing tau inclusions, and reduces neuronal demise in layers I–III of the cerebral cortex and the pontine nucleus of the brainstem [56], indicating that autophagy may also participate in tau degradation. In summary, autophagy plays a complex role in AD that must be further characterized to determine the potential contribution of autophagy to different stages and features of AD such as amyloidopathy.

Parkinson’s Disease (PD) is a neurodegenerative movement disorder that is characterized by the selective loss of substantia nigra pars compacta (SNpc) dopaminergic neurons, which results in a depletion of the neurotransmitter dopamine (DA) in the striatum [57]. Another feature of PD pathology is the presence of ubiquitylated α-synuclein-containing inclusions within neurons called Lewy bodies [57], suggestive of defects in intracellular protein clearance mechanisms. Indeed, autophagy failure is one of the pathophysiological mechanisms that has been linked to the initiation and progression of PD. Thus, mutations or polymorphisms that increase the susceptibility to suffer from sporadic PD or lead to familiar forms of PD usually affect the functionality of intracellular transport pathways that end up in the lysosome [58]. For instance, loss of function mutations in PTEN-induced putative kinase-1 (PINK-1) and Parkin, which cause sporadic juvenile-onset and autosomal recessive PD, respectively, regulate mitophagy in several in vitro and in vivo models [59]. Nevertheless, the physiological role of these proteins in nigral DAergic neurons and their actual contribution to neurodegeneration in PD still remains elusive [59]. On the other hand, brain tissue derived from sporadic PD patients exhibits an accumulation of autophagosomes together with a loss of lysosomal markers in affected DAergic neurons [60,61], suggesting a blockade of autophagy flux. Moreover, the expression of Transcription Factor EB (TFEB), a major regulator of autophagy and lysosomal biogenesis [62], is restricted to the cytoplasm in SNpc DA neurons of PD patients, whereas it is found in the cytoplasm and the nucleus of control brains [63], further indicating that the autophagic process may be dysregulated. In agreement with findings in human tissue, the disruption of autophagy flux has been confirmed using rodent models of PD [60,63], where α-synuclein load [63], DAergic neurodegeneration [60,63], and motor dysfunction [63] are significantly attenuated by the pharmacological or genetic upregulation of TFEB function. Overall, autophagy flux dysregulation seems to be a major contributor to PD neuropathology, having an impact on the development of α-synucleinopathy, SNpc DAergic neurodegeneration, and motor function.

Huntington’s Disease (HD) is an autosomal dominant inherited form of neurodegeneration caused by expansion of a CAG trinucleotide repeat in the first exon of the huntingtin gene. This expansion encodes a polyglutamine tract in the N-terminal region of the mutant huntingtin protein (mHTT), which contributes to its incorrect folding and aggregation in neurons, eventually leading to their death [64]. Huntingtin is highly expressed in neurons and participates in many cellular functions [65,66], including vesicle and organelle transport [67] and autophagy [68]. As such, several pathophysiological mechanisms have been associated with neurodegeneration in HD [69]. Interestingly, recent data indicate that wild-type HTT participates in autophagy, acting as a scaffolding protein [70] that facilitates substrate recognition by physically interacting with the autophagy machinery [71]. This physiological function of HTT matches with previously published data indicating that relatively empty autophagosomes are observed in mHTT-expressing cells and mouse models, as well as cells derived from patients with HD [72], suggesting defects in cargo loading into autophagosomes. Nevertheless, it is not clear whether clearance of autophagosomes in HD is accomplished normally [72] or whether mHTT prevents correct autophagosome transport and maturation along the neuronal axon, leading to a deficient fusion with lysosomes [73]. Of note, enhancing mHTT clearance through pharmacological [74,75,76] and genetic [77] interventions that may increase autophagy flux attenuates neurodegeneration [74,76,77] and improves motor performance [74,75,76] in mouse models of HD. In conclusion, a complex bidirectional relationship exists between mHTT and autophagy in HD [68]. Thus, the consensus in the literature is that mHTT interferes with the correct autophagic function, whereas enhancing the clearance of mHTT by autophagy relieves neurons from toxicity.

### 4.4. Autoimmunity-Mediated Secondary Neurodegeneration—Multiple Sclerosis

Multiple sclerosis (MS) is a chronic immune-cell-mediated disease of the CNS characterized by inflammation, demyelination, axonal and neuronal damage, and the formation of a glial scar [78]. Mechanistically, it is accepted that MS is an autoimmune disease wherein auto-reactive T cells target the myelin sheath in the CNS [79], secondarily leading to impairments in neuronal function. It has been shown that survival of auto-reactive T cells in a mouse model of experimental acute encephalomyelitis (EAE) depends on autophagy-related protein BECN-1 [80], suggesting a role for autophagy in the progression of MS. In agreement, ATG-5 expression is increased in circulating immune cells of mice with EAE, as well as T cells from blood and brain tissue of MS patients [81]. Additionally, several autophagy-related genes including ATG-16L2, ATG-9A, and ULK-1 are upregulated in blood samples of patients diagnosed with MS [82], which suggests that an overactivation of autophagy may underlie MS pathology through the modulation of T lymphocyte survival. On the other hand, ATG-7 modulates antigen presentation by dendritic cells (DCs), which influences T cell activation in EAE. Thus, DC-specific deletion of ATG-7 reduces antigen presentation by DCs to T cells and alleviates EAE pathology [83]. Additionally, treatment with the lysosomal inhibitor chloroquine before or after EAE onset delays progression or reduces the severity of disease, respectively [83], suggesting that the autophagy machinery may facilitate antigen presentation by DCs, eliciting T cell priming and autoimmunity in EAE, and possibly in MS. Overall, autophagy-related genes seem to affect DC-mediated activation of T cells as well as the survival of auto-reactive T lymphocytes, playing a deleterious role in MS pathology through the promotion of T cell-mediated demyelination and neurodegeneration.

In conclusion, important advances have been made in recent years to determine the role of autophagy in brain aging and neurodegeneration. However, additional efforts should be made to clarify the specific contribution of autophagy to different phases and types of brain diseases. Hence, the pro-survival effects of autophagy in neurons may be beneficial under certain conditions such as physiological aging, AD, PD, and HD, whereas the survival promoting effects of autophagy may have detrimental consequences for neuronal health in the context of T cell-mediated autoimmunity during MS. Similarly, autophagy may play a benign or a deleterious role in the context of harsh nutrient deprivation such as ischemia/stroke, possibly related to the variability of the autophagic response depending on the intensity of ischemic injury as well as the dynamics of recovery during the post-injury phase. Therefore, potential novel pharmacological approaches modulating autophagy in CNS aging and disease should consider variable outcomes arising from autophagy manipulation in different cell types (e.g., neurons, glial cells, and/or peripheral immune cells infiltrating the brain parenchyma) and contexts, which may account for different functional outcomes. In addition, therapies aimed at positively modulating autophagy should consider that autophagy may promote tumor survival and proliferation by sustaining cell metabolism [84], which may be a limitation for such strategies. In summary, further work is needed to choose the appropriate therapeutic strategy that may positively or negatively balance the autophagic response to maintain it at the cytoprotective range in each disease.

## 5. Autophagy in Microglia

Although most studies assessing the role of autophagy in neurodegeneration and aging have focused on neurons, emerging work suggests that autophagy may also contribute to glial cell function [85,86]. Here we will focus on microglia, the major player of the innate immune system in the brain. Certainly, microglia orchestrate the brain inflammatory response, responding to CNS damage and regulating the release of soluble inflammatory mediators as well as phagocytosing CNS-specific debris [87]. Mounting evidence indicates that autophagy finely regulates both innate and adaptive responses in the peripheral immune system [16], actively influencing the outcome of inflammatory responses [88], phagocytosis [89], and antigen presentation [90]. Of note, both immune senescence [91] and the low-grade chronic inflammation that characterizes aging [92] have been correlated with impaired autophagy flux in macrophages [93]. However, few studies have evaluated the role that autophagy may play on microglia and the possible impact of autophagy dysregulation on the physiology and survival of these brain-resident macrophages. In the next sections, we will review the studies that have assessed the role of autophagy in microglial phagocytosis and inflammation and try to open new research fields based on data coming from studies mainly performed in peripheral macrophages and microglia.

## 6. Autophagy and Microglial Phagocytosis

**Figure 1:**
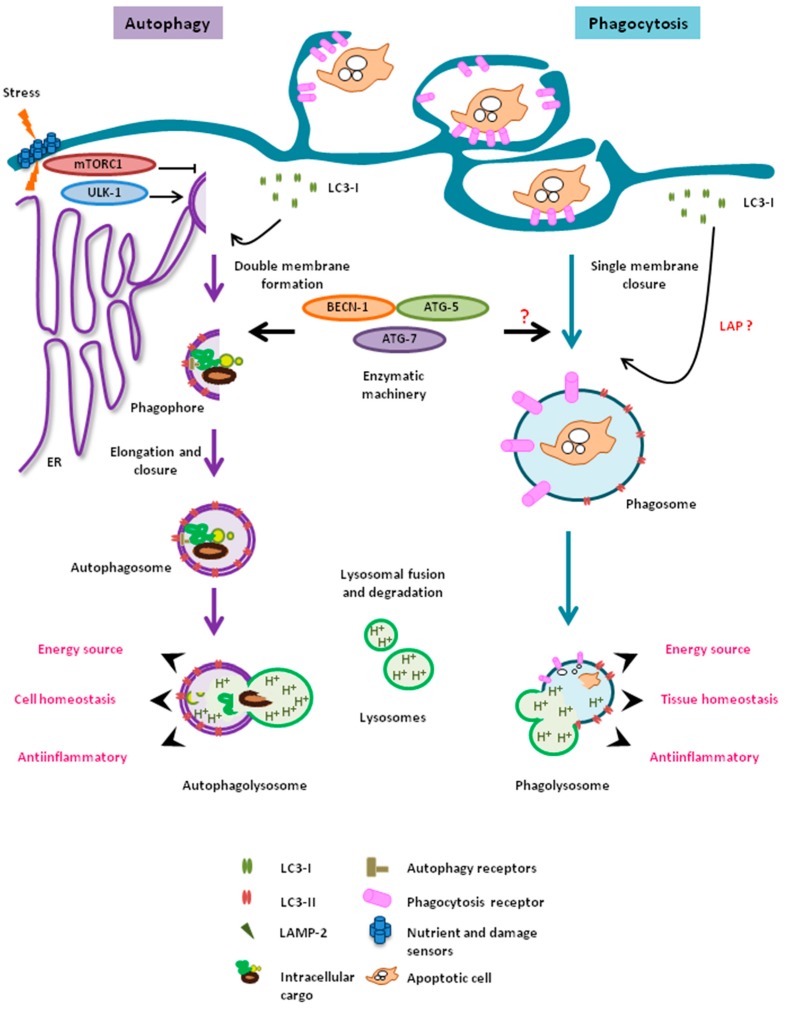
Autophagy and phagocytosis are lysosomal clearance pathways that share mechanistic and functional similarities. In response to cellular stress, autophagy (purple flow) is activated by signals that inhibit mechanistic target of rapamycin complex 1 (MTORC1) and activate unc-51 like autophagy activating kinase 1 (ULK-1), whereas phagocytosis (blue flow) is activated by extracellular ligands that bind to phagocytosis receptors in the surface of the microglial plasma membrane. Then, cargo engulfment structures start to form: the phagophore is de novo formed using the endoplasmic reticulum (ER) as a membrane source (autophagy) and the phagocytic cup is formed from invaginations of the plasma membrane (phagocytosis). These structures elongate and close up, forming the double-membrane-bound autophagosome (autophagy) and the single-membrane-containing phagosome (phagocytosis), which contain intracellular and extracellular degradative substrates, respectively. The formation of the autophagosome depends on the sequential and coordinated action of autophagy-related (ATGs) proteins, including microtubule-associated light chain 3 (LC3). In contrast, the formation of the phagosome may depend on the recruitment of autophagy machinery (ATGs and LC3) during LC3-associated phagocytosis (LAP) (described in peripheral macrophages, but not microglia; red question mark in the figure), or may be completed independently of ATGs in other types of phagocytosis. Finally, the autophagosome (autophagy) and the phagosome (phagocytosis), which contain the degradative cargo on their lumen, progressively mature and fuse with lysosomes, forming the autophagolysosome and the phagolysosome, respectively, where the cargo is finally digested. Autophagy and phagocytosis may serve similar functions in microglia, including the supply of energy during nutrient shortage, maintenance of cellular and tissue homeostasis, and the promotion of a net anti-inflammatory phenotype (see main text for details).

Microglia are the brain’s professional phagocytes, capable of engulfing and degrading microbes as well as different types of brain-derived cargo such as apoptotic cells, myelin and axonal fragments, protein deposits including Aβ, and synaptic material, among other substrates [87]. Phagocytosis is an evolutionarily conserved mechanism whereby a cell recognizes, engulfs, and degrades extracellular material in lysosomes utilizing a pathway that to some extent is similar to autophagy. Certainly, autophagy and phagocytosis share striking morphological and mechanistic similarities (Figure 1), as both processes rely on the formation of transient vesicular structures (autophagosomes and phagosomes, respectively) that engulf and deliver cargo to the lysosomes for digestion. Moreover, autophagy and phagocytosis are primitive forms of nutrient acquisition, and both processes have evolved to play essential functions in the maintenance of cellular and tissue homeostasis through the degradation of detrimental intra- and extra-cellular material, respectively. In contrast to autophagy, which can be found in virtually all cellular types and tissues in mammals [94], phagocytosis has mainly endured in a set of immune cells including macrophages and microglia, dendritic cells, and neutrophils [87]. Interestingly, recent evidence indicates that functional cross-talk may exist between autophagy and phagocytosis during the innate immune response in peripheral macrophages.

The potential regulatory action of autophagy over phagocytosis may occur at different steps of the phagocytic cascade, including cargo engulfment, maturation of phagosomes, and their fusion with lysosomes, which may affect phagocytic cargo uptake and/or degradation. In the next paragraphs, we will first summarize the evidence linking autophagy to phagocytic degradation efficiency in macrophages. Then, we will describe emergent studies suggesting other types of regulatory interactions between autophagy and phagocytosis. Notably, no studies have specifically assessed the role of autophagy in phagocytic uptake and/or degradation by microglia, and thus the section will be devoted to outline potential mechanisms that might occur in microglia.

### 6.1. LC3-Associated Phagocytosis

One of the most prominent examples of the cross-talk between autophagy and phagocytosis is the recent discovery of LC3-associated phagocytosis (LAP) in macrophages. During this process, the autophagy machinery is partially translocated to the phagosome to promote an efficient intracellular processing of the engulfed extracellular cargo. LAP has an impact on the subsequent immune response through the modulation of antigen presentation [90,95] and the regulation of the inflammatory profile [96,97,98]. Thus, upon Toll-like receptor (TLR)- or T cell immunoglobulin mucin protein 4 (TIM4)-stimulated phagocytosis of bacteria [99] or apoptotic cells [97] by macrophages, LC3 is recruited to the single-membrane phagosome depending on the activity of autophagy enzymes BECN-1, ATG-5, and ATG-7 [97,99] but not the recruitment of ULK-1 [97]. As a consequence, LC3 associates to single-membrane-bound phagosomes [97,99] but not to double-membrane-containing autophagosomes after phagocytosis [99], suggesting phagocytosis induction in the absence of an autophagic response. This novel process has been linked to the development of systemic lupus erythematosus (SLE) in vivo [98], a peripheral autoimmune disease associated with defects in the clearance of dead cells [89]. The dysregulation of LAP is associated with a net elevation of pro-inflammatory cytokines and increased levels of serum autoantibodies, which precipitates the appearance of a SLE-like phenotype [98].

During LAP, the recruitment of the autophagy machinery promotes the efficient degradation of extracellular cargo by phagocytosis. However, it is not clear whether the translocation of autophagy proteins to phagosomes is a sine qua non event for the completion of phagocytosis [100]. Indeed, a recent study has shown that macrophages derived from mice lacking ATG-5 and ATG-7 do not exhibit a delay in the maturation of opsonized particle- or zymosan-containing phagosomes to phagolysosomes [100], suggesting that autophagy proteins may not be required for all types of phagocytosis. In addition to the recruitment of some proteins of the autophagy machinery to the phagosome during LAP, a recent report has suggested that autophagy may be activated during phagocytosis of apoptotic cells in macrophages [101]. In this study, treatment with the autophagy inhibitor 3-MA had a deleterious impact on the viability and phagocytic function of macrophages [101], indicating a possible cytoprotective effect of autophagy over phagocytosis. However, 3-MA has been shown to inhibit LAP [98] and therefore it is not clear whether the effects observed on phagocytosis are a consequence of inhibition of either autophagy or LAP.

In conclusion, the discovery of LAP in peripheral macrophages has demonstrated that the autophagy machinery and phagocytosis intersect under certain conditions to promote proficient degradation of phagocytic cargo and, more importantly, has paved the road to elucidate the consequences of LAP dysregulation in physiology and disease. Nevertheless, no study has yet assessed whether LAP occurs in microglia and what the consequences of the lack or dysfunction of LAP may lead to in the CNS. As such, the potential contribution of microglial LAP to CNS physiology and pathology should be assessed in forthcoming years.

### 6.2. Autophagy Modulation of Phagocytosis Efficiency

In addition to LAP, the prior activation of autophagy may also modulate phagocytosis efficiency. Thus, autophagy flux-inducing stimuli such as nutrient starvation or rapamycin reversibly attenuate yeast phagocytosis by cultured peripheral macrophages [102], suggesting that autophagy induction may decrease the phagocytic capacity of macrophages. Nevertheless, this report did not provide conclusive evidence of whether autophagy flux was induced by nutrient starvation and rapamycin, nor whether phagocytosis was prevented through the regulation of the recognition, the internalization, or the degradation of phagocytic substrates. In contrast, another study has shown that nutrient deprivation enhances autophagy flux in macrophages, which in turn upregulates engulfment of bacteria [103]. Nevertheless, increased phagocytic uptake does not depend on autophagy flux induction since ATG-7 knockdown does not attenuate this response [103]. In conclusion, more studies are needed to clarify the consequences of autophagy activation on the phagocytic response.

Autophagy machinery may also regulate phagocytosis through the modulation of phagocytic receptor expression. For instance, the lack of ATG-7 in peripheral macrophages increases phagocytic uptake of bacteria, possibly through the enhancement of the expression of scavenger receptors in phagocyte cell surface [104]. Nonetheless, this study did not discard the possibility of LAP induction in macrophages, complicating the interpretation of data. In summary, further work is needed to thoroughly assess the impact of autophagy flux enhancement over phagocytosis, taking into consideration that LAP induction may be a possible confounding factor that may interfere with autophagy measurements. Of note, none of these studies were performed in microglia, and thus the impact of autophagy induction in microglial phagocytosis remains to be tested.

## 7. Autophagy and Microglial Phagocytosis in Aging and Neurodegeneration

Accumulating evidence indicates that impaired microglial function contributes to loss of CNS homeostasis during aging and age-related neurodegenerative diseases [105,106]. The mechanisms regulating microglial phagocytosis efficiency have mostly been studied in vitro, and only recently have we started to unravel the involvement of (dys)functional microglial phagocytosis in different brain disorders. For instance, microglial phagocytosis of apoptotic cells is chronically impaired in mouse and human mesial temporal lobe epilepsy [107]; aberrant phagocytosis of live neurons occurs during ischemia [108]; non-productive phagocytosis of Aβ may occur in AD [109,110,111]; and abnormal engulfment of synaptic terminals may underlie AD [112] and fronto-temporal dementia [113] pathology. In the next section, we will summarize the evidence linking autophagy to microglial phagocytosis of apoptotic cells, Aβ, synaptic material, and myelin debris.

### 7.1. Apoptotic Cells

Microglial removal of dying cells in the aging and damaged brain is a crucial mechanism to prevent the spillover of toxic molecules to the brain parenchyma, which avoids the appearance of autoimmunity as well as chronic inflammation, and promotes CNS homeostasis [87]. One example of this critical function is experimental and human mesial temporal lobe epilepsy, during which phagocytosis of hippocampal apoptotic cells by microglia is impaired by the hyperactivity of the neuronal network, resulting in delayed cell clearance and inflammation [107]. Similarly, genetic manipulations of phagocytosis in the fruit fly *Drosophila melanogaster* also demonstrate the impact of engulfment of apoptotic cells to aging and neurodegeneration. For instance, deletion of the phagocytic receptor Draper leads to developmental accumulation of apoptotic neurons, which persist undegraded throughout the lifespan and induce neurodegeneration with increased age [114]. Genetic screening of engulfment- and/or degradation-related phagocytic receptors indicates that glial phagocytic defects and the persistence of apoptotic bodies in *Drosophila* brain are associated with dysfunctional phagosome maturation rather than with impaired engulfment [114]. Moreover, phagocytosis in *Drosophila*’s brain may be similar to LAP described in macrophages, as TORC1 activation (a fruit fly protein homologous to mammalian MTORC1) or inhibition of ATG1 (a fruit fly protein homologous to mammalian ULK-1), which may inhibit autophagy flux, prevents apoptotic cell body induced neurodegeneration [114], suggesting that classical autophagy inhibition may enable translocation of the autophagosome formation machinery to LAP. In conclusion, these results suggest that autophagy inhibition may enable a LAP-like mechanism in invertebrate glial cells that promotes correct lysosomal processing of apoptotic cells and prevents neurodegeneration. Therefore, it is tempting to speculate that a similar mechanism may occur during phagocytosis of apoptotic cells by microglia in the degenerating brain. Nonetheless, no study has yet assessed the potential contribution of autophagy to phagocytosis of dead cells by microglia.

### 7.2. Amyloid-β

Microglia participate in the clearance of proteins with a high turnover rate as Aβ [87,111]. Interestingly, recent work has highlighted a role for the autophagy-related protein BECN-1 in Aβ phagocytosis by microglia. Genetic downregulation of BECN-1 reduces microglial Aβ uptake in vitro in culture and ex vivo in hippocampal brain slices containing Aβ deposits [115]. The reduction of Aβ load is impaired in the frontal cortex of mice with heterozygous deletion of BECN-1 [115] (homozygous deletion is embryonically lethal [116]), suggesting that BECN-1 may be necessary for Aβ internalization and/or degradation by microglia. In line with this finding, BECN-1 deficiency disrupts endocytic recycling of phagocytic receptors such as CD36 and Triggering Receptor Expressed on Myeloid cells 2 (TREM2) [115], which indicates that the autophagy-related protein BECN-1 impacts microglial Aβ phagocytosis through the regulation of cell surface expression of phagocytic receptors. Nonetheless, the effects of BECN-1 deletion in microglial autophagy flux and its possible impact in Aβ metabolism were not assessed in this study.

On the other hand, another study has suggested that Aβ fibrils may also be degraded by classical autophagy in microglia. Hence, extracellular Aβ fibrils are internalized by cultured microglia and disappear from the intracellular milieu in a time-dependent fashion [117], indicative of intracellular Aβ digestion by microglia. This degradation depends on microglial ATG-7 and LC3 activity since knockdown of these proteins prevents the clearance of Aβ [117]. Although these data have been interpreted as autophagic digestion of Aβ fibrils by microglia, this study did not provide evidence of how Aβ was internalized (e.g., endocytosis, phagocytosis, or other mechanisms) and did not discard the possibility that LAP-mediated clearance of Aβ may occur in microglia, which may also depend on ATG-7 and LC3 activity, similar to what occurs in peripheral macrophages [97,99]. Therefore, it has still not been clarified whether Aβ in microglia is degraded by classical autophagy or by a cooperative mechanism that involves both autophagy and phagocytosis such as LAP. Additionally, whether microglia efficiently engulf and degrade pathological Aβ deposits in vivo in rodent models of AD is a matter of controversy [111]. Altogether, more studies are needed to understand the relationship between microglial autophagy and phagocytosis in Aβ clearance and their potential regulation in pathological conditions such as AD.

### 7.3. Synaptic Pruning

Adult microglia may play a role in the refinement of synaptic connections in the mature CNS [118], similar to the role of microglia in synaptic pruning during development [119,120]. Indeed, emerging evidence indicates that microglial synaptic remodeling may be dysfunctional during neurodegenerative diseases such as AD [112] and fronto-temporal dementia (FTD) [113]. Thus, synapse loss in AD [112], which is an early feature of the disease that appears before Aβ plaque deposition and that correlates with cognitive decline [121], has been suggested to be a consequence of the aberrant engulfment of synapses by microglia [112]. Particularly, the presence of oligomeric Aβ induces an abnormal deposition of the complement effector C1q in AD synapses, which triggers the activation of the classical complement cascade and promotes dysfunctional microglial phagocytosis of synaptic material and synapse loss [112]. Of note, genetic silencing of complement effectors and receptors prevents AD-associated loss of synapses [112], indicating that the microglial-complement axis may affect AD synaptic pathology through dysfunctional phagocytosis. In agreement, a recent study has confirmed that a similar mechanism underlies synapse loss in a mouse model of FTD [113], which suggests that defective microglial synaptic pruning may be a common feature that contributes to the progression of different neurodegenerative diseases. Nevertheless, these studies did not show active engulfment of synapses by phagocytic microglia. Instead, they quantified the presence of synaptic markers inside microglial cells. Therefore, it cannot be excluded that microglia may have internalized and digested synaptic proteins and/or portions by alternative mechanisms such as endocytosis and autophagy, respectively.

As studies assessing the role of microglial synaptic pruning in neurodegeneration are currently emerging, the possible involvement of autophagy in refinement of synapses by microglia during aging and neurodegeneration has not been evaluated yet. However, it has recently been suggested that autophagy-related genes may play a role in the development of autism spectrum disorders (ASD) by influencing microglial synaptic pruning [122]. Thus, conditional deletion of ATG-7 from cells of myeloid origin elicits synaptic and brain wiring alterations, which have been correlated with the appearance of ASD-like behavioral abnormalities, including social defects and repetitive behaviors [122]. Nonetheless, ATG-7 is not exclusively involved in classical autophagy but it also affects LAP in peripheral macrophages [97,99]. Therefore, more studies are needed to dissect the possible contribution of autophagy to microglial synaptic pruning.

### 7.4. Myelin Debris

Myelination in the CNS is mainly carried out by oligodendrocytes [123], but myelin clearance by phagocytosis involves microglia and/or macrophages depending on context [87,123]. Recently, it has been reported that aged rodents exhibit a gradual increase in myelin breakdown fragments in the CNS [124], which has been correlated with reduced turnover of myelin debris by microglia in vivo [124]. Thus, age-associated brain demyelination is associated with an enlargement of the lysosomal compartment and the formation of lipofuscin-like insoluble aggregates containing myelin in microglial lysosomes [124]. Moreover, in vitro and in vivo models of demyelination display accelerated formation of lysosomal inclusions in microglia [124], suggesting that age-related excess myelin burden dysregulates lysosomal function in microglia. However, this study did not provide evidence of whether myelin clearance defects in microglia were a consequence of autophagy and/or phagocytosis dysregulation.

Indeed, peripheral nervous system (PNS) data indicate that a selective form of autophagy, myelinophagy, is involved in myelin digestion by Schwann cells after nerve injury [125,126]. Certainly, many components of the autophagy machinery including ULK-1, BECN-1, and ATG-7 are transcriptionally upregulated after sciatic nerve transection elicited demyelination [125] and LC3-II protein levels and green fluorescent protein (GFP)-LC3 puncta (autophagosome formation markers) are increased after axotomy in peripheral nerves [126]. Additionally, double-membrane-bound autophagosomes containing myelin debris are observed by electron microscopy in demyelinating Schwann cells from cultured nerve segments [125], and pharmacological (3-MA) or genetic (ATG-7 conditional knockout in Schwann cells) inhibitors of autophagy impair Schwann cell myelin digestion after nerve injury in vivo [125,126]. Altogether, these results indicate that autophagy in Schwann cells is involved in myelin digestion after peripheral nerve injury. Nevertheless, they do not discard the possibility that phagocytosis [127,128,129] and/or LAP may also play a role in Schwann cell-mediated myelin degradation. In addition, tissue resident macrophages including microglia may contribute to myelin clearance by phagocytosis [130,131]. Overall, the available data suggest that myelin may be digested by autophagy and/or phagocytosis depending on the cell type and its context. However, it remains to be elucidated what is the exact contribution of autophagy and/or phagocytosis to myelin clearance by microglia during aging and neurodegeneration.

In summary, accumulating evidence suggests that the autophagy machinery modulates phagocytosis in macrophages and microglia. Thus, autophagy genes may modulate phagocytic uptake through the regulation of the expression of surface engulfment receptors. Additionally, some autophagic enzymes contribute to phagocytic degradation through LAP, i.e., the translocation of some of its effectors to phagosomes to modulate their maturation into phagolysosomes. On the other hand, some data suggest that a functional overlap may exist between autophagy and phagocytosis. Thus, it is not clear whether extracellular substrates such as Aβ, myelin, and/or synaptic debris are cleared through autophagy, phagocytosis and/or by a cooperative action between the two processes in microglia. Notably, the discovery of LAP in macrophages has called into question the actual contribution of autophagy to extracellular substrate degradation in phagocytes. Accordingly, many of the pharmacological and genetic approaches used in autophagy research may also affect LAP, which complicates data interpretation. In conclusion, autophagy and phagocytosis seem to be intimately linked processes that may be reciprocally regulated in macrophages and microglia. As such, the accurate elucidation of the dynamic relationship between autophagy and phagocytosis in microglia during forthcoming years will surely advance our understanding of the role of microglial (dys)function in healthy aging and disease.

## 8. Autophagy and Microglial Inflammation

Microglia are the main immune effectors of the brain [132] and efficiently respond to CNS injury, dynamically adapting their inflammatory phenotype to the changing environment [133]. Although the microglial inflammatory response is acutely initiated to neutralize harm and to promote tissue repair and functional recovery, the sustained activation of the inflammatory reaction due to unresolved damage is associated with loss of CNS homeostasis and neurotoxicity [134]. As such, innate immunity mediated inflammation is a tightly regulated process at different cellular levels, including ligand binding, signal transduction, transcription, and epigenetics, whose outcome ultimately depends on context-associated balance of pro- and anti-inflammatory cytokines as well as other inflammatory mediators [135]. Notably, proteolytic pathways such as the ubiquitin-proteasome system also contribute to the regulation of inflammation [135,136]. In agreement, recent work indicates that autophagy controls the inflammatory response in innate immune macrophages [16,137] and microglia [138].

### Autophagy and Inflammasomes

Inflammasomes are cytosolic macromolecular complexes that are assembled after stimulation by infectious or damaging stimuli, and regulate the activity of inflammatory proteases of the caspase family [139,140]. Diverse types of inflammasomes have been identified, and the assembly of each type depends on the driving stimulus [139]. Nonetheless, all types of inflammasomes contain a ligand sensor and an adaptor protein known as Apoptosis-associated Speck-like protein containing CARD (ASC), which bridges the inflammasome sensor to caspase-1 upon activation, and leads to the maturation of pro-inflammatory cytokines interleukin-1β (IL-1β) and interleukin-18 (IL-18) [139,140].

**Figure 2:**
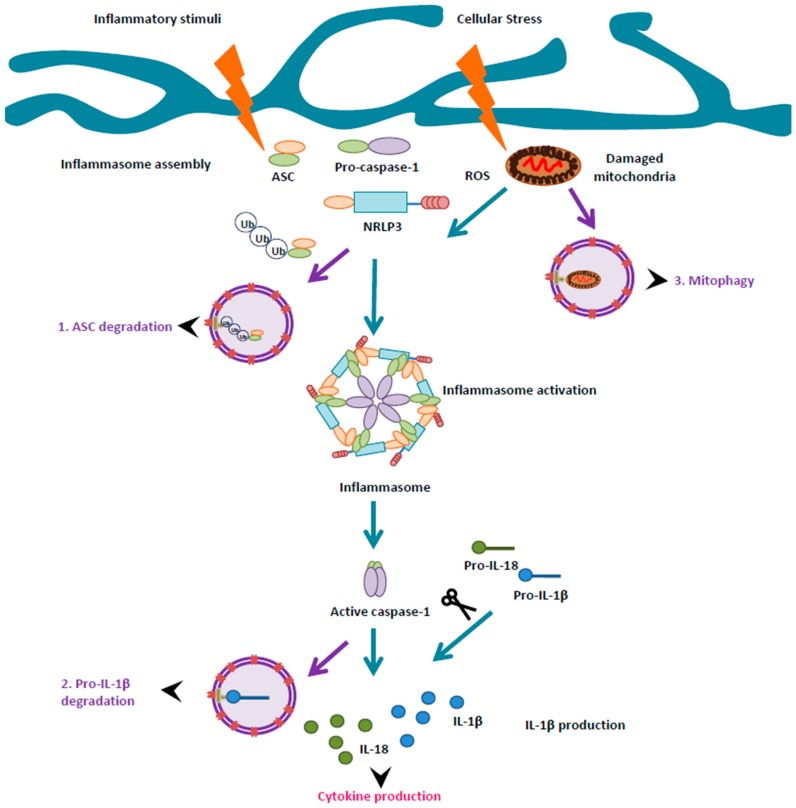
Autophagy may negatively regulate microglial inflammation: potential mechanisms of inflammasome regulation. Inflammasomes are cytosolic macromolecular sensors that assemble after activation by infectious or damaging stimuli (illustrated by blue arrows). They consist of a ligand sensor (i.e., NLRP3), an adaptor protein (ASC), and the immature form of the inflammatory caspase, pro-caspase-1. Inflammasome assembly induces the proteolytic processing of pro-caspase-1 to its active form caspase-1. Subsequently, activated caspase-1 proteolytically processes the immature forms of inflammatory cytokines pro-IL-1β and pro-IL-18 to active inflammatory mediators IL-1β and IL-18. In peripheral macrophages, three types of modulatory interactions have been described to explain the suppresive effect of autophagy over the inflammasome (illustrated by purple arrows). Thus, autophagy may target (1) the inflammasome adaptor protein ASC and/or (2) the inflammasome substrate pro-IL-1β for digestion to the lysosome. On the other hand, autophagy may (3) selectively digest damaged, ROS-generating mitochondria by mitophagy. Note that none of these mechanisms have yet been described in microglia (see main text for details).

Inflammasome activation is regulated by several mechanisms, such as the stimulation by pathogen- and/or danger-associated molecular patterns, which are related to infectious agents and stressful stimuli, respectively [139,140]. Recent work has demonstrated that autophagy suppresses the activation of inflammasomes [16,137]. Indeed, autophagy flux impairments enhance inflammasome-dependent production of IL-1β in mouse models of peripheral inflammatory diseases such as Crohn’s disease [141] and atherosclerosis [142]. Conversely, reduced inflammasome activity has been observed after treatment with compounds that may activate autophagy flux in peripheral models of inflammatory disease [16], suggesting that autophagy may provide protection against chronic inflammation. The mechanisms whereby autophagy may inactivate the inflammasome are varied (Figure 2), including the preoteolytic clearance of its adaptor protein ASC and/or its substrate pro-IL-1β, as well as through selective targeting of damaged, radical oxygen species (ROS) generating mitochondria, to the lysosome through mitophagy [16]. In agreement with findings in macrophages, emerging data indicates that autophagy also negatively regulates inflammasomes in microglia [138], including during Aβ-induced neuroinflammation in vivo [117] and EAE [143], which will be detailed in the section below.

## 9. Autophagy and Microglial Inflammation during Aging and Neurodegeneration

There is increasing evidence that microglia-driven release of pro-inflammatory mediators substantially impacts brain injury during ischemia/stroke [144], chronic neurodegeneration in AD, PD, and HD [48,106,145,146], as well as promoting autoimmunity-mediated MS pathology [134,147]. Moreover, the microglial inflammatory response leads to the infiltration of peripheral immune effectors to the brain, which further contribute to the inflammatory release. Indeed, the peripheral immune system drives demyelination in MS [79], sustains inflammation, or promotes repair depending on context during ischemic damage [144] and may also contribute to late stages of AD, PD, and HD [148,149]. In the next paragraphs, we will summarize the evidence linking autophagy dysregulation in microglia with inflammation in neurodegeneration and aging.

### 9.1. Aging

Aging is associated with a macrophage- and/or microglia-driven low-grade chronic inflammation known as “inflamm-aging” in peripheral tissues as well as the CNS [92,150,151], which predicts the vulnerability to suffer from neurodegenerative diseases such as AD [152]. Certainly, microglia derived from the brains of aged mice display increased basal levels of pro-inflammatory cytokines Tumor Necrosis Factor-α (TNF-α), IL-1β, and Interleukin-6 (IL-6) as well as anti-inflammatory mediators IL-10 and Transforming Growth Factor-β (TGF-β) [153]. The stimuli that induce and sustain inflammation during aging seem to arise from undigested cellular debris (e.g., damaged cells and organelles) as well as altered self-molecules [92], suggesting that aging may be related to the impairment of lysosomal clearance mechanisms such as autophagy and/or phagocytosis. Accordingly, a recent study has suggested that autophagy gene deficiency is related to the appearance of an age-related inflammatory phenotype in peripheral macrophages. Thus, ATG-7 lacking macrophages exhibit enhanced inflammasome activity and increased levels of pro-inflammatory cytokines IL-1β, IL-6, granulocyte-macrophage colony-stimulating factor (GM-CSF), and TNF-α under basal and bacterial lypopolysaccharide (LPS) stimulated conditions [93]. These results indicate that loss of ATG-7 induces a pro-inflammatory profile in macrophages, similar to the age-associated loss of autophagy flux in aged macrophages [93]. However, no studies have yet evaluated the role that autophagy may play in age-associated microglial inflammation.

### 9.2. Acute Neurodegeneration—Ischemia/Stroke

The microglial inflammatory response contributes to all stages of adult ischemic stroke [154,155]. Hence, brain ischemia acutely elicits a pro-inflammatory microglial phenotype that enhances neurotoxicity whereas at later stages microglia orchestrate a beneficial trophic response to promote repair and tissue regeneration [154,155]. Interestingly, recent reports indicate that autophagy may modulate microglial inflammation in rodent models of brain ischemia. Accordingly, mice subjected to permanent medial cerebral artery occlusion (pMCAO) display a time-dependent increase in pro-inflammatory cytokines TNF-α, IL-1β, and IL-6 in the cerebral cortex [156]. This effect may be autophagy-dependent since treatment with 3-MA, which decreases cortical LC3-II levels, prevents this response [156]. Conversely, treatment with rapamycin enhances cortical levels of these inflammatory mediators [156], although the effects of rapamycin on microglial autophagy flux were not assessed in this study. Overall, these results suggest that autophagy may modulate the microglial inflammatory response during hypoxia/ischemia. Nevertheless, as whole tissue homogenates from cerebral cortex were used to perform these assays, the contribution of other CNS resident cells such as astrocytes, endothelial cells, and/or infiltrating immune cells to the inflammatory response cannot be excluded. Similarly, 3-MA microinjection in the ischemic hemisphere inhibits the inflammation associated [157] transcription factor nuclear factor kappa B (NFκB) pathway and decreases TNF-α and IL-6 levels in rats subjected to focal cerebral ischemia [158]. In contrast, inhibition of the glycogen synthase kinase-3β (GSK-3β) reduces IL-1β, TNF-α, and inducible nitric oxide synthase (iNOS) after pMCAO in rats [159]. These effects have been attributed to the stimulatory effects of the GSK-3β inhibitor over LC3-II levels, an autophagosome formation marker, in microglia isolated from pMCAO-subjected rats [159]. Altogether, these studies suggest that autophagy may modulate the microglial inflammatory response after ischemic brain injury, although it is not clear whether autophagy positively or negatively regulates microglial inflammation.

### 9.3. Chronic Neurodegeneration—Alzheimer’s, Parkinson’s, and Huntington’s Diseases

Chronic inflammation is a common hallmark of neurodegenerative diseases such as AD, PD, and HD [106,145]. Emerging data indicate that autophagy may play a role in the modulation of the microglial inflammatory response during AD and PD. However, no studies have yet assessed whether autophagy is involved in microglial inflammation in HD.

In AD, autophagy seems to regulate Aβ-mediated inflammasome activation in microglia. Thus, LC3 or ATG-7 knockdown induces activation of the nucleotide-binding domain, leucine-rich-repeat containing, pyrin-domain-containing 3 (NLRP3) inflammasome and enhances IL-1β secretion in fibrillar Aβ-treated cultured microglia [117]. In contrast, LC3 knockdown does not affect TNF-α release by microglia [117], suggesting that the autophagy machinery may selectively modulate the NLRP3 inflammasome in microglia challenged with Aβ. Interestingly, conditioned media from LC3 silenced microglia induce neuronal damage in vitro, whereas compounds that may activate autophagy flux significantly prevent inflammation and neurotoxicity [117], which indicates that autophagy-related genes LC3 and ATG-7 play a role in the regulation of Aβ-induced inflammasome activation in microglia. In agreement, conditional ATG-7 deletion in cells of myeloid origin exacerbates inflammation in the hippocampus of fibrillar Aβ injected mice, concomitantly elevating caspase-1 and IL-1β levels [117], suggesting that the autophagy machinery may also regulate Aβ-elicited inflammasome activation in microglia in vivo in AD.

Emerging studies in PD also suggest that autophagy may regulate microglial inflammation. Indeed, intra-SN infusion of 1-methyl-4-phenylpyridinium (MPP^+^) in rats enhances active caspase-1 and cathepsin B levels in nigral microglia, which correlate with increased IL-1β in SN [160]. Interestingly, the anti-inflammatory phenolic flavonoid, baicalein, attenuates inflammation and up-regulates LC3-II levels in the SN of MPP^+^ injected rats [160], suggesting that autophagy modulation regulates the microglial inflammatory response in PD. Nevertheless, this study did not clarify whether MPP^+^ elicited increases in LC3-II levels were associated with activation or blockade of autophagy flux, and thus it is inconclusive on the possible contribution of autophagy to microglial inflammation in PD. Another recent study has also shown that metformin, an MTORC1 inhibitor currently used to treat type 2 diabetes, may activate autophagy flux in the SN of 1-methyl-4-phenyl-1,2,3,6-tetrahydropyridine/probenecid (MPTPp) mouse model of PD. Metformin attenuates inflammasome activation and pro-inflammatory cytokine TNF-α and IL-6 levels while increasing anti-inflammatory IL-10 levels [161], suggesting that autophagy may control the SN inflammatory response in an in vivo model of PD. However, this report did not provide evidence of the specific role of microglia on MPTPp elicited PD-like inflammatory response. As such, further studies are needed to unravel the potential involvement of autophagy in PD-related microglial neuroinflammation.

### 9.4. Autoimmunity-Mediated Secondary Neurodegeneration—Multiple Sclerosis

In MS, autophagy also seems to play a role in microglial inflammation. Thus, treatment with a cannabinoid receptor 2 (CB2R) agonist increases expression of autophagy proteins BECN-1 and LC3-II while suppressing NLRP3 inflammasome activation in the spinal cord of mice with EAE, which correlates with improved clinical and histological score [143]. Notably, in vitro experiments using a microglial cell line confirm that the CB2R agonist enhances these autophagy markers in an ATG-5-dependent manner, and prevents inflammasome activation and the subsequent production of IL-1β [143]. Taken together, these results suggest that the autophagy machinery in microglia may regulate the inflammatory response underlying EAE.

Overall, emergent studies suggest that autophagy may prevent inflammasome activation and promote a net anti-inflammatory phenotype of microglia during aging and neurodegenerative disease, but further studies are needed to confirm the context- and phase-specific influence of autophagy dys(regulation) to chronic neuroinflammation.

## 10. Concluding Remarks

Autophagy is emerging as a crucial mechanism that impacts neuronal and glial health through the modulation of lysosomal clearance mechanisms as well as by contributing to the proteolytic processing of cell signaling mediators such as inflammatory cytokines. Recent data suggest that reduced autophagic function may accompany physiological brain aging and indeed impaired autophagy flux has extensively been described in neurons of animal models of age-related neurodegenerative diseases, including ischemia/stroke, AD, PD, and HD (Table 1). On the other hand, autophagy machinery seems to promote the activation and survival of auto-reactive T cells in MS, promoting demyelination and neurodegeneration. As such, the pro-survival effects of autophagy may be beneficial in the setting of primary neurodegeneration (i.e., AD, PD, HD) but detrimental during autoimmunity-mediated secondary neurodegeneration (i.e., MS). Additionally, it has still not been clarified whether excess autophagy and/or autophagy flux dysregulation contributes to neurotoxicity during ischemia/stroke. Until recently, autophagy research in the CNS has mainly focused on neurons, without considering the potential consequences of autophagy (dys)regulation in glial cells, including microglia. Of note, recent discoveries point to autophagy as a substantial regulator of innate immune responses such as phagocytosis and inflammation in peripheral macrophages. In agreement, emerging evidence suggests that autophagy modulation in microglia may have functional consequences on microglial phagocytosis and inflammation, which may contribute to the progression of neurodegeneration and brain aging (Table 1). Therefore, we believe that during the next decade autophagy research in microglial cells will lead to exciting discoveries that will substantially expand our knowledge of the pathophysiological mechanisms driving CNS degeneration and aging. Moreover, the elucidation of cell-type-specific mechanisms governing autophagy and its impact on overall brain physiology will aid in the exploration and development of new autophagy-centered therapeutic strategies that may considerably benefit public health.

## Figures and Tables

**Table 1:** Summary of the role that autophagy plays in neurons and microglia during aging, ischemia/stroke, AD, PD, and HD. This table briefly describes autophagy impairments described in neurons, the outcome of the pharmacological or genetic modulation of autophagy in animal models of neurodegeneration and aging, and the impact of autophagy dys(regulation) in microglial phagocytosis and inflammation in the context of CNS senescence and disease. Note that many of the effects of autophagy (dys)regulation in microglial function are still unknown. Multiple Sclerosis, as a T cell-mediated demyelinating disease that exhibits no intrinsic neuronal damage, has not been included in the table (see main text for details). References are in parenthesis. PD: Parkinson’s Disease, HD: Huntington’s Disease, AD: Alzheimer’s Disease, I/S: ischemia/stroke.

Disease	Neurons		Microglia			
	Autophagy		Phagocytosis		Inflammation	
	Status	Role	Function	Role of autophagy	Function	Role of autophagy
**Aging**	Downregulation of autophagy genes and proteins [30,31,32]. Increased MTORC1 activity [31,32].	Autophagy inhibition (by ATG-5/7 deletion) results in spontaneous neurodegeneration [27,28]. Autophagy activation (by caloric restriction) prevents brain atrophy and enhances learning/memory [32,35].	Clearance of apoptotic cells, Aβ, synaptic material, and myelin debris [87].	Autophagy inhibition (by MTORC1 activation or ATG1 inhibition) prevents neurodegeneration [114].	Inflamm-aging [92,150,151,153].	Unknown.
**PD**	Blockade of autophagy flux [60,61].	Autophagy activation (by TFEB activation) reduces neurodegeneration and improves motor performance [60,63].	Clearance of apoptotic cells [87].	Unknown.	Inflammatory mediator production [106,160,161].	Baicalein increases LC3-II expression and attenuates inflammation [160]. Autophagy activation (MTORC1 inhibition) reduces inflammatory mediators [161].
**HD**	Defects in autophagosome loading [72] and/or maturation [73].	Autophagy activation (by MTORC1 inhibition and others) reduces neurodegeneration and improves motor performance [74,75,76,77].	Clearance of apoptotic cells [87].	Unknown.	Inflammatory mediator production [145].	Unknown.
**AD**	Defects in autophagy flux [51,52,53].	Autophagy activation (by cystatin b deletion) reduces Aβ load, and reduces learning/memory deficits [54]. Autophagy inhibition (by ATG-7 deletion) reduces extracellular Aβ deposition, and increases learning/memory deficits [55]. Autophagy activation (by trehalose) decreases tau inclusions and neurodegeneration [56].	Clearance of apoptotic cells, synaptic debris, and Aβ [87,109,110,111,112].	Autophagy inhibition (by BECN-1 downregulation) reduces Aβ phagocytosis and/or degradation [115]. Autophagy inhibition (by ATG-7 and LC3 deletion) reduces Aβ clearance [117].	NLRP3 inflammasome activation and inflammatory mediator production [117].	Autophagy inhibition (by LC3 and ATG-7 deletion) activates NLRP3 inflammasome and inflammatory mediator production [117].
**I/S**	Increased autophagy markers [40,41] and mitophagy [46].	Autophagy activation (by MTORC1 inhibition) decreases neurodegeneration [42,43]. Autophagy (by TSC1 deletion and 3-MA) and mitophagy (by mdivi-1) inhibition increases neurodegeneration [44,46]. Autophagy inhibition (by chloroquine, 3-MA, and BECN-1 deletion) decreases neurodegeneration [41,43,47].	Clearance of apoptotic cells [87]. Phagocytosis of live cells [108].	Unknown	Inflammatory mediator production [154,155].	GSK-3β blockade increases LC3-II expression and reduces inflammatory mediator release [159].

## Abbreviations

CNS	Central nervous system
MTORC1	Mechanistic target of rapamycin 1
ULK-1	Unc-51 like autophagy activating kinase 1
BECN-1	Beclin-1
Vps34	Vacuolar protein sorting 34
PI3P	Phosphatidyl-inositol-3-phosphate
ATG	Autophagy-related gene
LC3	Microtubule-associated light chain 3
SQSTM-1	Sequestosome 1
NBR1	Neighbor of BRCA1
NIX	NIP-3 like protein X
NCCD	Nomenclature committee on cell death
3-MA	3-methyladenine
PI3K	Phophatidyl-inositol-3-kinase
CR	Caloric restriction
PD	Parkinson’s Disease
SNpc	Substantia nigra pars compacta
DA	Dopamine
PINK-1	PTEN-induced putative kinase-1
TFEB	Transcription factor EB
HD	Huntington’s Disease
mHTT	Mutant huntingtin
HTT	Huntingtin
AD	Alzheimer’s Disease
Aβ	amyloid-β
PS-1	Presenilin-1
TSC-1	Tuberous sclerosis complex-1
mdivi-1	Mitochondrial division inhibitor-1
tMCAO	Transient middle cerebral artery occlusion
MS	Multiple sclerosis
EAE	Experimental autoimmune encephalomyelitis
DC	Dendritic cell
LAP	LC3-associated phagocytosis
TLR	Toll-like receptor
TIM4	T cell immunoglobulin mucin protein 4
SLE	Systemic lupus erythemathosus
TREM-2	Triggering receptor expressed on myeloid cells 2
FTD	Frontotemporal dementia
ASD	Autism spectrum disorders
PNS	Peripheral nervous system
GFP	Green fluorescent protein
ASC	Apoptosis-associated speck-like protein containing CARD
ROS	Radical oxygen species
IL-1β	Interleukin-1β
IL-18	Interleukin-18
IL-6	Interleukin-6
GM-CSF	Granulocyte-macrophage colony stimulating factor
TNF-α	Tumor necrosis factor-α
IL-10	Interleukin-10
TGF-β	Transforming growth factor-β
LPS	Lipopolysaccharide
NLRP3	nucleotide-binding domain, leucine-rich-repeat-containing, pyrin-domain-containing 3
MPP^+^	1-Methyl-4-phenylpyridinium
MPTPp	1-Methyl-4-phenyl-1,2,3,6-tetrahydropyridine/probenecid
CB2R	Cannabinoid receptor 2
pMCAO	Permanent middle cerebral artery occlusion
GSK-3β	Glycogen synthase kinase-3 β
iNOS	Inducible nitric oxide synthase
